# Unveiling Myoepithelioma: A Case Report and Literature Review of an Uncommon Soft Tissue Tumor

**DOI:** 10.1002/ccr3.71177

**Published:** 2025-10-05

**Authors:** Abdul Moiz Khan, Syed Wajihullah Shah, Qazi Muhammad Safwan, Rida Akhtar, Jibran Ikram, Muhammad Abu Bakkar

**Affiliations:** ^1^ Pakistan Institute of Medical Sciences Islamabad Pakistan; ^2^ Khyber Medical College Peshawar Pakistan; ^3^ Hayatabad Medical Complex Peshawar Pakistan; ^4^ Cleveland Clinic Foundation Cleveland Ohio USA; ^5^ Khyber Teaching Hospital Peshawar Pakistan

**Keywords:** case report, myoepithelioma, neoplasm, parotid gland tumor, salivary gland

## Abstract

Myoepitheliomas are rare salivary gland tumors, comprising only 1%–1.5% of all cases. We report a unique case of a 69‐year‐old woman with a painless, slowly enlarging right parotid mass. CT imaging showed an ill‐defined, irregular, multilobulated heterogeneous lesion compressing the deep parotid lobe. Fine‐needle aspiration cytology suggested a benign spindle cell tumor. The patient underwent superficial parotidectomy, and histopathology confirmed a benign myoepithelioma with spindle and epithelioid cells, along with distinctive fibro‐myxoid changes. Immunohistochemistry was positive for p63 and S‐100. Postoperative recovery was uneventful, and no recurrence was noted at two‐year follow‐up. This case highlights the importance of considering myoepithelioma in elderly patients and demonstrates distinct radiological and histological features aiding diagnosis.

## Graphical abstract

Upper Frame: Hematoxylin and Eosin (H&E) stained sections showing histopathological features of myoepithelioma involving the parotid gland in low‐power view (10×). (A) Low‐power view (10×) highlighting benign neoplasm composed of spindle to epithelioid cells (white arrow). (B) Similar low‐power view (10×) highlighting spindle to epithelioid cell proliferation (white arrow) with replacement of normal parotid glandular architecture (black arrow). Lower Frame: Hematoxylin and Eosin (H&E) stained sections showing histopathological features of myoepithelioma involving the parotid gland in high‐power view (40×). A: High‐power view (40×) highlighting the clear cell component (white arrows) in addition to spindle cells (black arrows). B: High‐power view (40×) highlighting myoepithelioma cells with a clear cell component (white arrows) along with spindle cells (black arrows).

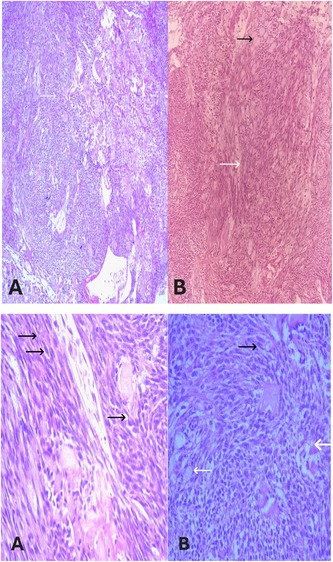

**Summary**

- Myoepithelioma of the parotid gland, though rare and typically seen in younger adults, should also be considered in elderly patients presenting with painless parotid swellings.
- Distinct radiological features and fibro‐myxoid histology can aid in diagnosis, and surgical excision remains curative with excellent outcomes.

## 1 Introduction

Myoepitheliomas are rare neoplasms of the salivary glands, accounting for approximately 1%–1.5% of all salivary gland tumors [1]. The majority of reported cases have been benign, with only a small proportion demonstrating pathological features suggestive of malignancy [2]. Their distribution among the salivary glands includes the parotid (48%), submandibular (10%), and minor salivary glands (42%) [3]. Due to their rarity and overlapping clinical and imaging features, myoepitheliomas can be challenging to distinguish from other benign salivary gland tumors, particularly pleomorphic adenomas. However, unlike pleomorphic adenomas, myoepitheliomas do not exhibit chondroid or osteoid formation [4].

Most reported cases occur in younger and middle‐aged adults, with only rare pediatric presentations [5]. The present case extends this spectrum to elderly patients, indicating that age alone should not exclude myoepithelioma from the differential diagnosis. Radiologically, computed tomography (CT) demonstrated an ill‐defined and irregular lesion compressing the deep parotid lobe, features that may mimic malignancy [6]. Histopathologically, the tumor demonstrated fibro‐myxoid changes in addition to spindle and epithelioid cell morphology, thereby broadening the recognized histological spectrum, which predominantly includes epithelioid and mixed cell types, with occasional spindle and other morphologies [7].

We report a rare case of parotid gland myoepithelioma in a 69‐year‐old woman, highlighting its atypical presentation in an elderly patient along with its distinct radiological and histopathological features.

## 2 Case Presentation/Examination

A 69‐year‐old woman from Pakistan presented with a painless nodule in the right parotid gland that had been gradually increasing in size over the past 6 months. She had no history of smoking, allergies, or other systemic conditions, except for osteoporosis. On clinical examination, an oval subcutaneous mass measuring 4 × 5 × 6 cm was palpated in the right parotid gland region. The mass was firm, elastic, slightly movable, and not associated with any cervical lymphadenopathy. Cranial nerve function was intact.

## 3 Methods

### 3.1 Differential Diagnosis

Given the patient's presentation, the differential diagnosis included pleomorphic adenoma and Warthin's tumor.

### 3.2 Investigations

**FIGURE 1:**
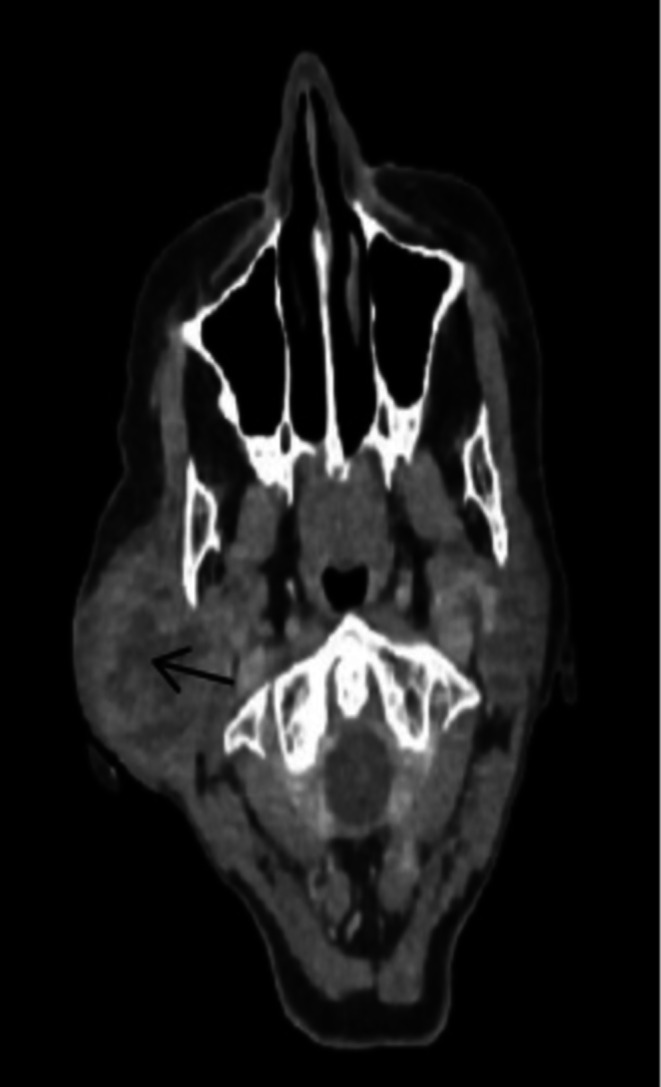
CT scan revealing an ill‐defined, irregular, multilobulated heterogeneous mixed‐density lesion originating from the right parotid gland.

A CT scan revealed an ill‐defined, irregular, multilobulated heterogeneous mixed‐density lesion originating from the right parotid gland and compressing its deep portion (Figure 1). Fine‐needle aspiration cytology (FNAC) showed monotonous spindle to epithelioid cells with no significant pleomorphism or mitosis, suggestive of a benign spindle cell tumor.

### 3.3 Treatment

Based on imaging and cytology findings, the multidisciplinary team opted for a right superficial parotidectomy. The procedure was conducted under general anesthesia using a standard modified Blair incision. Intraoperative facial nerve monitoring was employed throughout to ensure nerve preservation. The surgical dissection was meticulous, prioritizing identification and preservation of the facial nerve branches. No intraoperative complications were encountered, and hemostasis was achieved. A drain was placed and removed 48 h postoperatively.

**FIGURE 2:**
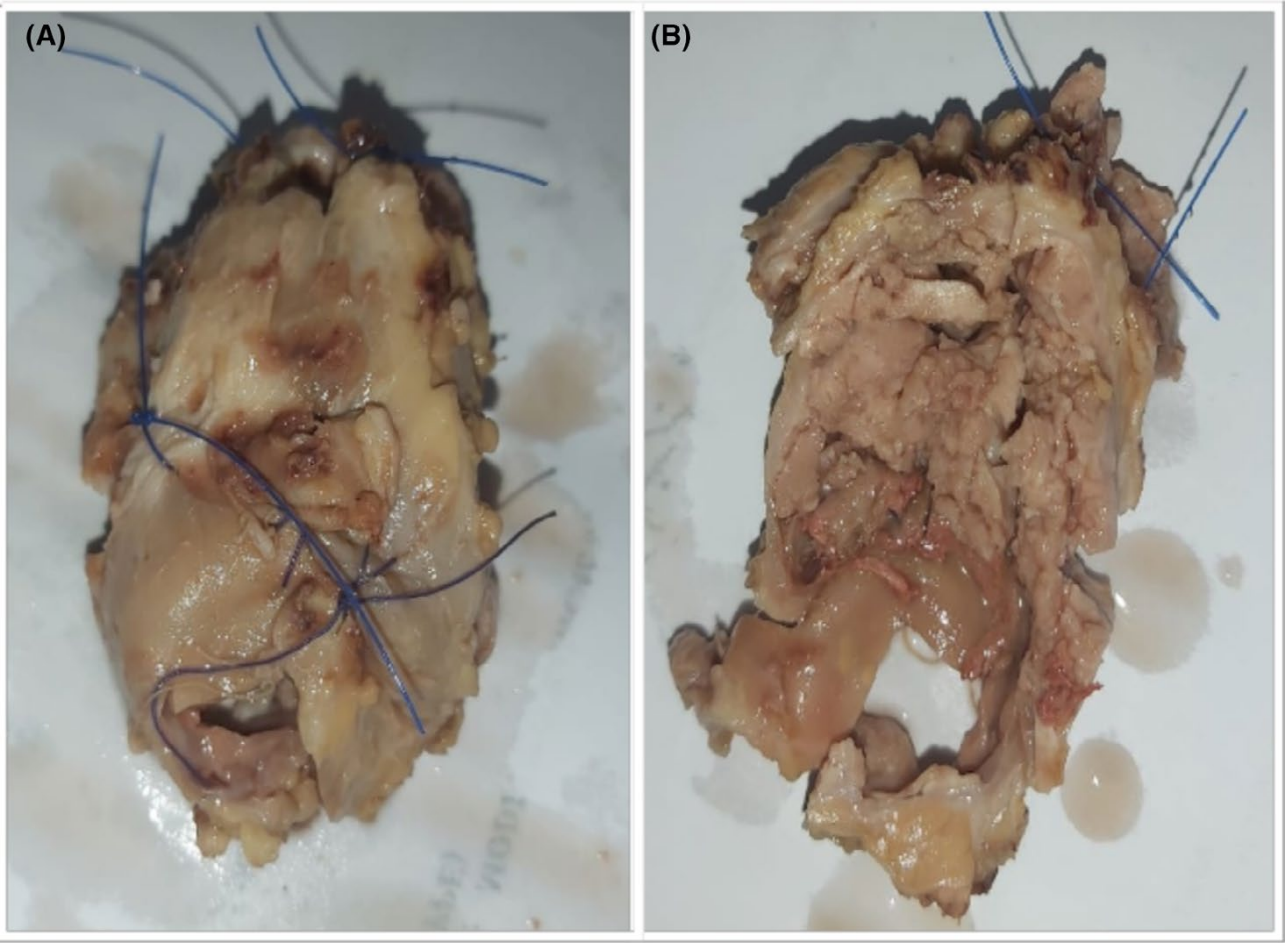
Superficial parotidectomy showing a well‐encapsulated whitish‐brown mass (A); cut section reveals a friable tumor replacing the normal parotid architecture (B).

Macroscopically, the tumor appeared as a well‐encapsulated and solid whitish‐brown mass (Figure 2A). On the cut section, it was friable and had replaced the normal parotid gland structure (Figure 2B).

**FIGURE 3:**
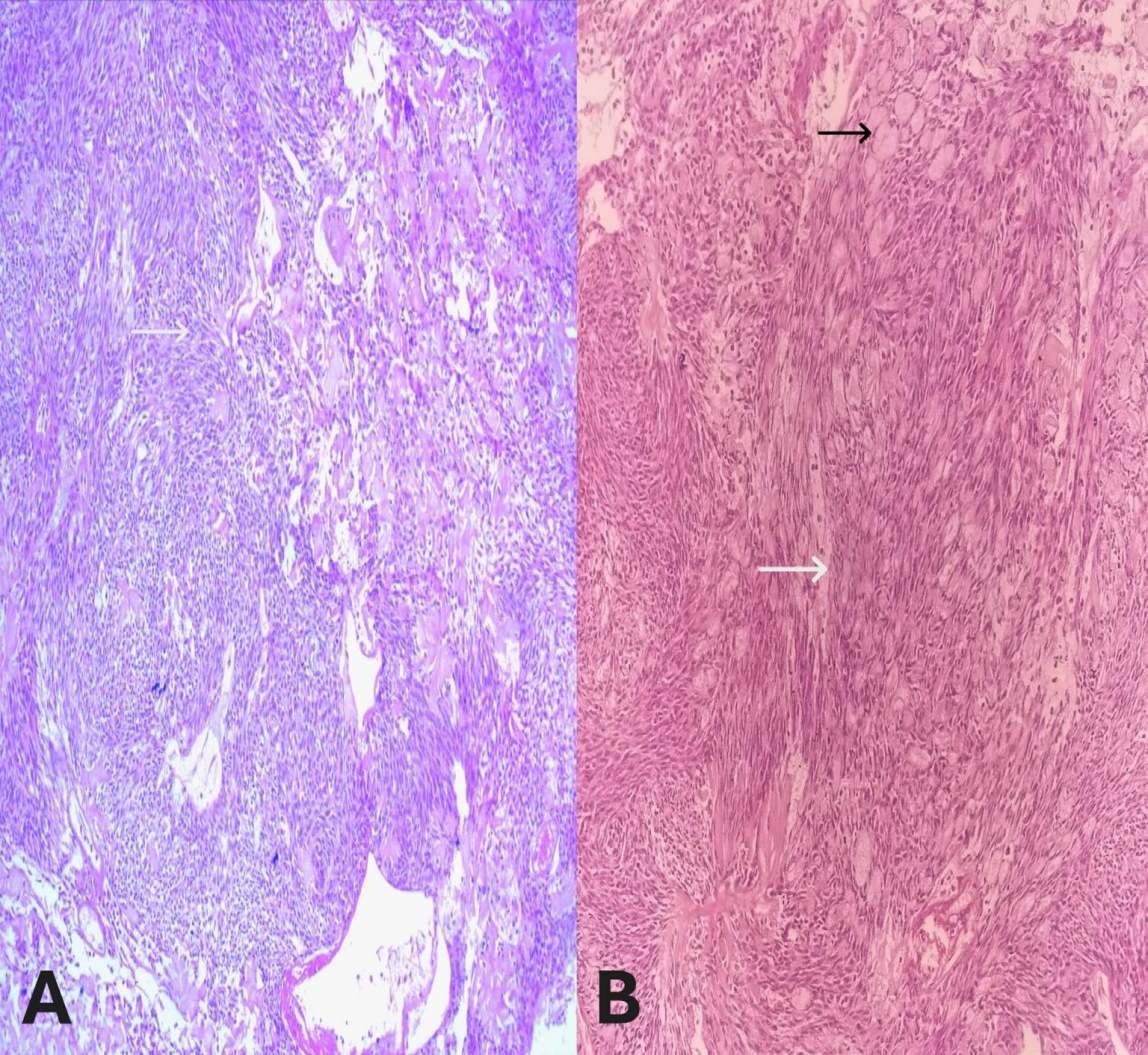
Hematoxylin and Eosin (H&E) stained sections showing histopathological features of myoepithelioma involving the parotid gland in low‐power view (10×). (A) Low‐power view highlighting benign neoplasm composed of spindle to epithelioid cells (white arrow). (B) Low‐power view highlighting spindle to epithelioid cell proliferation (white arrow) with replacement of normal parotid glandular architecture (black arrow).

No pathological cervical lymphadenopathy was observed. The specimen was sent for definitive histopathological analysis. Histopathological examination confirmed a well‐circumscribed and encapsulated benign neoplasm composed of spindle to epithelioid cells with ample cytoplasm and focal fibro‐myxoid changes with few foci showing normal parotid glandular component (Figure 3A,B).

**FIGURE 4:**
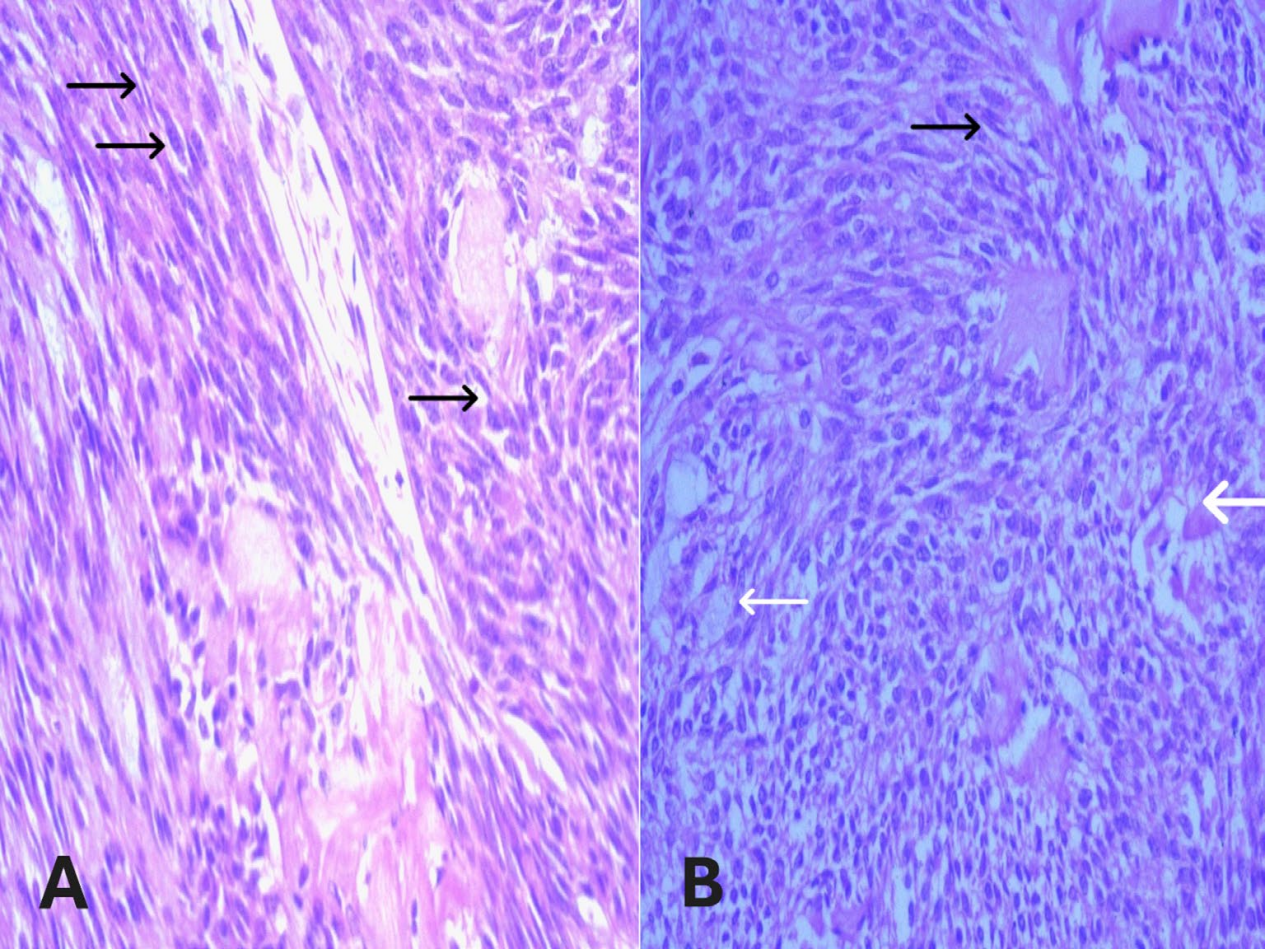
Hematoxylin and Eosin (H&E) stained sections showing histopathological features of myoepithelioma involving the parotid gland in high‐power view (40×). (A) High‐power view highlighting the clear cell component (white arrows) in addition to spindle cells (black arrows). (B) High‐power view highlighting myoepithelioma cells with a clear cell component (white arrows) along with spindle cells (black arrows).

The tumor cells had abundant clear cytoplasm, with no evidence of coagulative necrosis or mitotic figures (Figure 4A,B).

**FIGURE 5:**
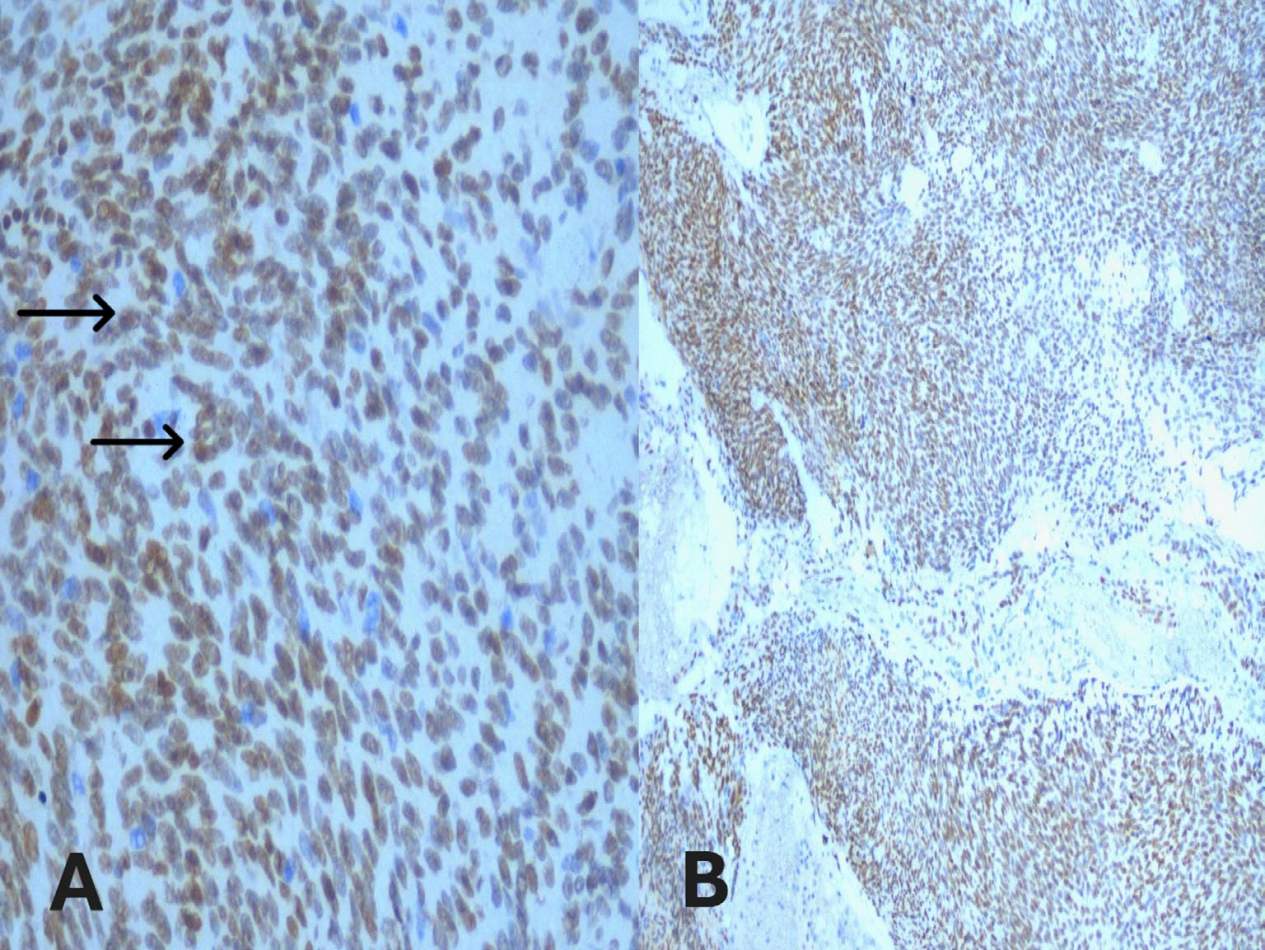
Immunohistochemical staining for p63 demonstrating nuclear positivity in myoepithelioma. (A) High‐power view (40×) showing strong nuclear p63 immunoreactivity in neoplastic spindle cells (black arrows). (B) Low‐power view (10×) highlighting diffuse nuclear p63 expression throughout the tumor cell population.

**FIGURE 6:**
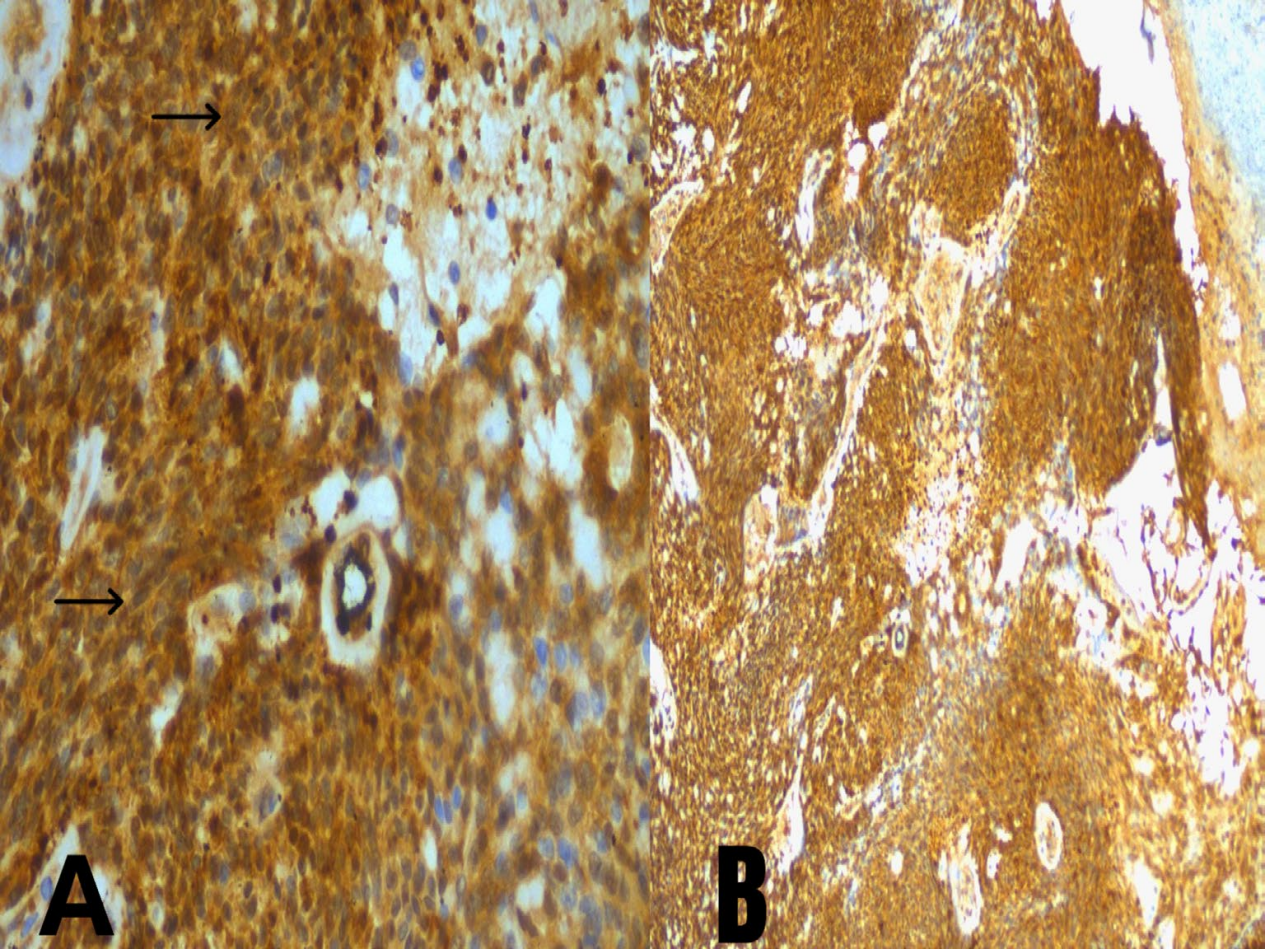
Immunohistochemical staining for S100 demonstrating diffuse positivity in myoepithelioma cells. (A) High‐power view (40×) showing strong nuclear and cytoplasmic S100 immunoreactivity in neoplastic spindle cells (black arrows). (B) Low‐power view (10×) highlighting diffuse and uniform S100 expression throughout the tumor.

Immunohistochemically, the neoplastic myoepithelial cells showed strong positivity for p63 (Figure 5A,B) and S‐100 (Figure 6A,B), confirming the diagnosis of myoepithelioma.

## 4 Conclusion and Results (Outcome and Follow‐Up)

The patient had an uneventful postoperative recovery, with preserved facial nerve function. She was educated about the signs of recurrence, including new swelling, pain, or facial weakness, and was advised regular self‐examination. Follow‐up imaging was not immediately warranted due to clear histology and margins but was planned if clinical suspicion arose. Regular follow‐ups were scheduled every 6 months. At the two‐year review, she remained asymptomatic with preserved facial function and no recurrence. The surgical scar was barely visible, and she reported complete symptom resolution.

## 5 Discussion

Myoepithelial cells are specialized contractile cells found primarily in the major and minor salivary glands, where they play a key role in glandular secretion. They have also been observed in other exocrine tissues, although their distribution varies. Notably, myoepithelial cells are absent in the pancreas [8, 9]. The World Health Organization first recognized myoepitheliomas as a distinct histological entity and classified them as a type of salivary gland tumor in 1991 [9, 10]. Myoepithelioma is an uncommon benign tumor of the salivary glands, composed mainly of myoepithelial cells. These cells can display a variety of shapes and organizational patterns, and the lesion generally presents as a well‐defined or encapsulated mass [1]. Myoepithelial tumors most commonly arise in adults between their 30s and 50s, affecting both genders equally. While these tumors can also develop in children, myoepithelial carcinomas are more prevalent than myoepitheliomas in the pediatric population. Affected individuals typically present with a palpable mass, which may or may not be accompanied by pain [11].

Myoepithelioma cells generally show immunopositivity for cytokeratins and vimentin. Vimentin is commonly expressed in neoplastic myoepithelial cells but not in their normal counterparts. Malignant transformation may lead to changes or loss of their myoepithelial (smooth muscle) characteristics [12].

Spindle cell myoepitheliomas typically demonstrate positive immunohistochemical staining for S‐100 protein, cytokeratins, calponin, smooth muscle actin (SMA), p63, and glial fibrillary acidic protein (GFAP). Among these, calponin is considered one of the most sensitive markers for detecting myoepithelial differentiation. Due to variability in marker expression, especially in tumors with diverse cellular morphology, the use of a combination of immunomarkers—including epithelial markers (such as cytokeratins), myogenic markers (like SMA and calponin), and S‐100 protein—is recommended for accurate diagnosis [13].

A comprehensive literature review of the past decade revealed a few reported cases of parotid gland myoepithelioma, highlighting its diverse clinical presentations, histopathological features, and outcomes [3, 13, 14, 15, 16, 17]. Most cases presented as slow‐growing, painless parotid masses, though some exhibited rapid growth or pain, raising suspicion for malignancy. Mughal et al. and Shenoy et al. [3, 14] described cases in younger patients with well‐demarcated tumors and benign histology, whereas Kalra et al. [17] noted an intermittently painful lesion. Oh and Moon [13] reported a rapidly growing tumor with necrosis, while Caon et al. [15] documented a malignant myoepithelioma with lymphadenopathy and inoperability, necessitating palliative radiotherapy. Weitzel et al. [9] observed a cystic presentation, and Soberanis‐Piña et al. [16] described an aggressive course with eventual metastasis. Immunohistochemistry findings varied, with S‐100, p63, and cytokeratin being the most expressed markers. Surgical excision remained the primary treatment, with prognosis largely favorable except in malignant cases. These cases are summarized in Table 1.

**TABLE 1:** Summary of reported cases of parotid gland myoepithelioma in the past decade.

Study	Age/sex	Symptoms/duration	Imaging findings	Treatment	Histopathology	IHC findings	Recurrence
Caon et al. [15]	87/M	Rapidly growing left parotid mass with necrotic cervical lymphadenopathy	Not mentioned	Palliative radiotherapy (45 Gy + 10 Gy boost), inoperable (T3N3M0)	Malignant myoepithelioma	Positive: S‐100, muscle actin	No recurrence at 38 months
Weitzel et al. [9]	NR	Slowly enlarging, painless left posterior auricular mass (5 years)	Multilobulated, cystic parotid tumor, no lymphadenopathy	Left superficial parotidectomy	Epithelioid myoepithelioma, fascicular pattern of neoplastic myoepithelial cells	Positive: Calponin, CK5/6, GFAP, p63, S‐100, CK7, vimentin, SMA; Negative: CK20	No recurrence at 10 months
Oh et al. [13]	53/M	Rapidly growing right parotid mass (1 year)	Well‐marginated, lobulated tumor, no lymph node involvement	Superficial parotidectomy	Spindle cell‐type myoepithelioma with necrosis	Positive: Vimentin, S‐100, pancytokeratin (focal), p63 (focal); Negative: Ema, Actin, desmin, CD34, ALK; Ki‐67 < 10%	No recurrence at 3 years
Piña et al. [16]	36/F	Painful parotid mass	Not mentioned	Superficial parotidectomy	Plasmacytoid myoepithelioma	Not mentioned	Progression to metastatic myoepithelial carcinoma (18/55 nodes, Ki‐67: 10%) treated with surgery, radiotherapy (66 Gy), and chemo (carboplatin‐paclitaxel); lung metastases at 10 months
Mughal et al. [14]	30/F	Painless right preauricular mass (6 months)	Not mentioned	Superficial parotidectomy	Myoepithelioma	Positive: Cytokeratin AE1/AE3, S‐100, ASMA	NR
Shenoy et al. [3]	10/M	Painless, slowly enlarging right parotid swelling (6 months)	Well‐demarcated, solid mass in the superficial lobe, no infiltration	Superficial parotidectomy	Myoepithelioma, mixed subtype (spindle and clear cell)	Not mentioned	NR
Kalra et al. [17]	42/F	Progressively enlarging, intermittently painful right infra‐auricular swelling (2 months)	Suggestive of benign parotid tumor	Right superficial parotidectomy with facial nerve preservation	Myoepithelioma (round to polygonal cells with eosinophilic cytoplasm)	Positive: CK7, p63; Negative: S‐100	No recurrence

Abbreviations: ALK, anaplastic lymphoma kinase; ASMA, alpha smooth muscle actin; CK, cytokeratin; EMA, epithelial membrane antigen; F, female; FNAC, fine‐needle aspiration cytology; GFAP, glial fibrillary acidic protein; Gy, Gray; IHC, immunohistochemistry; Ki‐67, proliferation index marker; M, male; NR, not reported; SMA, smooth muscle actin.

In summary, our case aligns with previously reported benign parotid myoepitheliomas in terms of painless, slow‐growing presentation, benign FNAC findings, and successful treatment with superficial parotidectomy. Histopathologically, it shares features with spindle and epithelioid subtypes, yet stands out due to fibro‐myxoid changes. Additionally, our case provides a more detailed radiological characterization, with CT imaging revealing an ill‐defined, irregular, multilobulated, heterogeneous mixed‐density lesion compressing the deep parotid gland, a feature not commonly emphasized in previous reports. Unlike malignant cases, there was no recurrence at two years. Given the older age (69 years) compared to most benign cases, our report highlights the importance of considering myoepithelioma in the differential diagnosis of parotid tumors in elderly patients.

## Author Contributions

**Abdul Moiz Khan:** conceptualization, data curation, formal analysis, investigation, methodology, project administration, resources, software, supervision, validation, visualization, writing – original draft. **Syed Wajihullah Shah:** conceptualization, data curation, formal analysis, investigation, methodology, project administration, resources, software, supervision, validation, visualization, writing – original draft, writing – review and editing. **Qazi Muhammad Safwan:** conceptualization, data curation, investigation, methodology, project administration, resources, software, supervision, validation, visualization, writing – original draft. **Rida Akhtar:** conceptualization, data curation, methodology, project administration, resources, software, supervision, validation, visualization, writing – original draft. **Jibran Ikram:** conceptualization, data curation, methodology, resources, software, supervision, validation, visualization, writing – original draft. **Muhammad Abu Bakkar:** conceptualization, data curation, investigation, methodology, project administration, resources, software, supervision, validation, visualization, writing – original draft.

## Ethics Statement

Ethical approval for this case report was obtained from the relevant institute, and written informed consent was provided by the patient in accordance with the journal's guidelines. The authors declare no additional ethical concerns.

## Consent

Written informed consent was obtained from the patient to publish this report in accordance with the journal's patient consent policy.

## Conflicts of Interest

The authors declare no conflicts of interest.

## Data Availability

Data supporting the conclusions of this report is contained within the report. Additional nonrelevant patient data are protected under patient privacy regulations and policies.
